# Association between the albumin–bilirubin score and gallstone prevalence: evidence from NHANES and an independent hospital-based cohort

**DOI:** 10.3389/fmed.2026.1872983

**Published:** 2026-09-10

**Authors:** Qi Sun, Xizhuang Gao, Hanning Zhang, Yuanjian Song

**Affiliations:** 1 Department of Endocrinology, Affiliated Hospital of Nantong University, Nantong University, Nantong, China; 2 Department of Functional Examination, Zaozhuang Municipal Hospital, Jining Medical University, Zaozhuang, China; 3 Department of Cardiology, Zaozhuang Municipal Hospital, Jining Medical University, Zaozhuang, China; 4 Department of Hepatology, Zaozhuang Municipal Hospital, Jining Medical University, Zaozhuang, China

**Keywords:** ALBI score, cross-sectional survey analysis, gallstones, hepatic function, machine learning

## Abstract

**Objective:**

This study aimed to evaluate the association between the albumin–bilirubin (ALBI) score and gallstone prevalence, and to develop machine-learning-based classification models for identifying participants with gallstones.

**Methods:**

Data from NHANES 2017–March 2020, including adults with complete albumin, bilirubin, and gallstone-status information, were analyzed. Weighted multivariable logistic regression and restricted cubic spline models were used to examine the association between ALBI score and gallstone prevalence. Fifteen feature-selection and machine-learning combinations were implemented to build classification models, with external validation conducted in a hospital-based cohort. Model performance was assessed using ROC curves, calibration plots, and decision curve analysis.

**Results:**

A total of 5,090 participants were included, including 490 with gallstones (9.63%, crude proportion). In the fully adjusted survey-weighted logistic regression model, continuous ALBI score was positively associated with gallstone prevalence (OR = 2.89; 95% CI: 1.68–4.99; *P* = 0.002). Restricted cubic spline analyses, stratified by sex, revealed a significant nonlinear association in women (*P* = 0.004) but not in men (*P* = 0.261), with a positive dose–response relationship when the ALBI score exceeded −2.60. In the machine-learning analysis, the leading cross-validated AUC values ranged from 0.718 to 0.720 across the top configurations, with internal test-set AUC values of 0.703 to 0.708; these differences were within the expected variability of the cross-validation procedure and the models should be regarded as indistinguishable in discriminative performance. In the external validation cohort, the AUC values ranged from 0.701 to 0.710, but the confidence intervals around these estimates are wide (approximately 0.62–0.80) owing to the small number of events (*n* = 42), and the external results provide only preliminary support for generalizability.

**Conclusion:**

In a national sample of US adults, a higher ALBI score remained associated with higher odds of gallstone disease after multivariable adjustment. Machine-learning models incorporating ALBI achieved moderate discrimination (cross-validated AUC up to 0.720; internal test AUC up to 0.708), with preliminary external-validation support that requires confirmation in larger cohorts.

## Introduction

The liver plays a vital role in maintaining systemic homeostasis and overall metabolic health. As one of the largest and most complex organs in the human body, it performs essential functions including metabolic regulation, detoxification, blood volume control, immune modulation, and the storage and transformation of nutrients (1, 2). The liver and gallbladder are physiologically interconnected components of the hepatobiliary system, working synergistically in the digestion, absorption, and metabolism of dietary fats (3). The liver produces bile, which is stored and concentrated in the gallbladder, and subsequently released into the small intestine following food intake—particularly fatty meals—to facilitate lipid digestion (4, 5).

Gallstones form primarily through the crystallization of bile components such as cholesterol or bilirubin when bile becomes supersaturated (6). The balance of bile composition, largely dependent on hepatic function, is essential for preventing gallstone formation. Impaired liver function—whether due to cirrhosis, steatosis, or viral hepatitis—can alter bile secretion and composition, thereby increasing susceptibility to gallstone disease and other gallbladder pathologies (3, 7).

The albumin–bilirubin (ALBI) score, introduced in 2015, is a simple and objective index for assessing liver functional reserve. Originally proposed to predict prognosis in patients with hepatocellular carcinoma (8), the ALBI score has since been applied in chronic viral hepatitis and cirrhosis (9–13), and in prognostic stratification in gastrointestinal malignancies such as pancreatic, colorectal, and gastric cancers (14–16). Furthermore, studies have linked ALBI to outcomes in cardiovascular conditions, including idiopathic dilated cardiomyopathy and heart failure treated with cardiac resynchronization therapy (17, 18). Despite its broad clinical utility, the association between the ALBI score and gallstone presence has rarely been investigated. Because bile secretion and composition depend in part on hepatic function, exploring this relationship may provide insight into the hepatobiliar*y* axis and metabolic determinants of gallstone disease.

In the present study, we aimed to evaluate the association between ALBI score and gallstone prevalence using data from the National Health and Nutrition Examination Survey (NHANES). Beyond traditional regression analyses, we incorporated machine-learning-based classification models to explore whether ALBI and related clinical variables could help identify individuals with gallstones. The leading models were also evaluated in an independent hospital-based cohort to assess their performance in a different clinical population. This study evaluated whether the ALBI score could serve as an accessible indicator associated with gallstone status.

## Methods

### Study design and population

This study adopted a dual-database design, comprising a primary analysis based on data from the National Health and Nutrition Examination Survey (NHANES) and an external validation using an independent hospital-based cohort. The NHANES database (2017–March 2020) is a nationally representative survey conducted by the U.S. Centers for Disease Control and Prevention (CDC), integrating demographic, dietary, clinical, laboratory, and questionnaire data from participants across the United States. All NHANES protocols were approved by the National Center for Health Statistics Research Ethics Review Board, and all participants provided written informed consent prior to participation (19).

For the present analysis, adults aged 18 years or older were eligible because the gallstone questionnaire item was administered to adult participants. We initially identified 15,560 participants from NHANES 2017–March 2020. We excluded 6,328 participants younger than 18 years and 22 participants with missing, refused, or “don't know” responses for gallstone status. Among the remaining 9,210 adults with complete gallstone-status data, we further excluded 4,120 participants who were pregnant or breastfeeding or had missing data on serum albumin, total bilirubin, poverty income ratio, education level, smoking status, alcohol consumption, diabetes, hypertension, or cardiovascular disease. Finally, 5,090 participants were included in the complete-case analytic sample. The participant-selection process is shown in Supplementary Figure S1.

To further evaluate the consistency of the findings and model performance in an independent clinical setting, we included a hospital-based retrospective cohort from Zaozhuang Municipal Hospital, Zaozhuang, China, covering the period from January 2021 to December 2025. Consecutive eligible participants meeting the inclusion criteria during the study period were enrolled until the target sample size was reached; no random selection or sampling fraction was applied. Eligible participants were adults aged 18 years or older with available serum albumin and total bilirubin measurements and clearly documented gallstone status based on abdominal ultrasonography or intraoperative findings. The cohort included adults who underwent abdominal ultrasonography as part of routine clinical care and had serum albumin and total bilirubin measured on the same admission. Participants were excluded if they had chronic liver disease, including viral hepatitis, liver cirrhosis, or hepatocellular carcinoma; malignancy; or incomplete clinical data.

After applying the eligibility and exclusion criteria, 500 participants were included in the hospital-based cohort, including 42 participants with gallstones and 458 participants without gallstones. The gallstone prevalence in the hospital cohort was 8.4%. Missing data were handled using complete-case analysis, and no imputation was performed. The baseline characteristics of the hospital-based cohort are presented in Supplementary Table S2.

The same ALBI formula and cutoff values were applied in both the NHANES and hospital cohorts. Predictors and preprocessing steps were aligned as closely as possible across the two data sources, with remaining differences documented in the mapping table (Supplementary Table S3). For external validation, the same core predictors used in the NHANES machine-learning pipeline were applied to the hospital cohort, including age, sex, race/ethnicity (a single constant category in the external cohort), socioeconomic indicator, education level, smoking status, alcohol consumption, diabetes, hypertension, and ALBI score.

### Definition of gallstones

In the NHANES cohort, gallstone status was determined using the standardized Medical Conditions Questionnaire item asking: “Has a doctor or other health professional ever told you that you had gallstones?” A response of “Yes” was defined as a self- or proxy-reported physician-diagnosed history of gallstones, whereas a response of “No” was defined as no reported history of gallstones. Participants with missing, refused, or “don't know” responses were excluded from the analysis. It should be noted that NHANES did not include abdominal ultrasonography or other imaging-based confirmation of gallstones.

In the hospital-based validation cohort, gallstone status was determined through abdominal ultrasonography or intraoperative findings recorded in the electronic medical record system, which provided imaging- or procedure-based confirmation of gallstone status.

### Definition and calculation of ALBI

The ALBI score was calculated using two objective biochemical parameters: serum total bilirubin and serum albumin levels. The formula is as follows:

ALBIscore=0.66×log10(totalbilirubin[μmol/L])−0.085×albumin[g/L].

Serum total bilirubin and albumin were expressed in the required SI units before calculation, with total bilirubin measured in μmol/L and albumin measured in g/L. The ALBI score was originally developed to quantify liver functional reserve, particularly in patients with hepatocellular carcinoma. According to the established classification system:

- Grade 1: ALBI ≤ −2.60
- Grade 2: −2.60 < ALBI ≤ −1.39
- Grade 3: ALBI > −1.39

In this study, because only one participant fell into Grade 3, Grades 2 and 3 were combined for statistical robustness. Therefore, the −2.60 cut-point, derived from the ALBI grading system for hepatocellular carcinoma, is used here solely as a pre-specified analytic cut-point to define the upper range of the score distribution, and it should not be interpreted as a validated clinical threshold in a community population.

- Low-range ALBI (ALBI ≤ −2.60)
- Upper-range ALBI (ALBI > −2.60)

The same formula, units, and cutoff points were applied consistently in both the NHANES and hospital cohorts to maintain comparability.

### Covariates

This study incorporated a comprehensive set of covariates to control for potential confounding factors influencing the relationship between the ALBI score and gallstone prevalence. Covariates were selected based on biological plausibility, clinical relevance, and previous literature, and were defined as consistently as possible across the NHANES and Chinese hospital-based cohorts, with differences in source definitions documented where applicable. Demographic and socioeconomic factors included age, sex, ethnicity, education level, and socioeconomic status, represented by the PIR in the NHANES dataset and by predefined income bands in the hospital cohort. In NHANES, ethnicity was classified as Non-Hispanic White, Non-Hispanic Black, Mexican American, Other Hispanic, and Other (including Asian and multiracial participants); whereas in the hospital cohort, all participants were of Asian ethnicity and thus treated as a homogeneous category. Educational attainment was categorized into two levels: high school or below and above high school. PIR in NHANES was categorized as low (<1.3), medium (≥1.3–<3.0), and high (≥3.0), while predefined income bands were used in the hospital cohort as the corresponding socioeconomic indicator. Lifestyle factors included smoking status and alcohol consumption. Smoking status was categorized as never, former, or current smoker. Alcohol consumption was categorized into four categories: never, mild, moderate, and heavy drinking. A mapping table (Supplementary Table S3) shows the correspondence between the alcohol consumption and socioeconomic categories across the two cohorts and identifies where the definitions differ. In NHANES, never drinking was defined as no alcohol consumption; mild drinking was defined as up to 1 drink/day for women and up to 2 drinks/day for men; moderate drinking was defined as 2–3 drinks/day for women and 3–4 drinks/day for men; and heavy drinking was defined as ≥4 drinks/day for women and ≥5 drinks/day for men. In the Chinese hospital cohort, alcohol intake was assessed using structured questionnaires and converted to grams of pure alcohol per day, and was categorized as never drinking (0 g/day), mild drinking (≤12 g/day), moderate drinking (>12–36 g/day), and heavy drinking (>36 g/day). Clinical comorbidities included diabetes mellitus, hypertension, and CVD. Diabetes was defined as self-reported physician diagnosis, current use of insulin or oral hypoglycemic agents, fasting plasma glucose ≥ 7.0 mmol/L, or HbA1c ≥ 6.5%. Hypertension was defined as systolic blood pressure ≥ 140 mmHg, diastolic blood pressure ≥ 90 mmHg, or use of antihypertensive medication. CVD encompassed coronary heart disease, heart failure, myocardial infarction, stroke, and angina pectoris. Variables were mapped across databases as closely as possible for the multivariable regression and machine-learning analyses, with remaining differences documented in Supplementary Table S3.

### Statistical analysis

All statistical analyses were performed using R software version 4.3.2, and a two-tailed *P* value < 0.05 was considered statistically significant. Owing to the complex, multistage sampling design of NHANES, sampling weights, masked variance pseudo-strata, and pseudo-primary sampling units were incorporated to obtain nationally representative estimates. Specifically, because gallstone status was obtained from questionnaire data and the ALBI score was calculated from MEC laboratory blood-test variables, the NHANES 2017–March 2020 pre-pandemic cycle full-sample MEC examination weight (WTMECPRP) was used together with SDMVSTRA and SDMVPSU. Survey weights, strata, and clusters were applied to descriptive statistics, multivariable logistic regression models, restricted cubic spline analyses, and subgroup analyses. Unweighted sample counts and survey-weighted percentages are reported where appropriate.

Continuous variables were summarized as weighted means with standard errors, and categorical variables were summarized as unweighted counts and survey-weighted percentages. Differences in baseline characteristics between participants with and without gallstones were evaluated using survey-weighted tests.

To examine the association between the ALBI score and gallstone prevalence, three multivariable logistic regression models were constructed. Model I was unadjusted. Model II was adjusted for age, sex, race/ethnicity, and education level. Model III was further adjusted for poverty income ratio, smoking status, alcohol consumption, diabetes, hypertension, and cardiovascular disease. The ALBI score was analyzed both as a continuous variable and as a categorical variable using the pre-specified cut-point of −2.60. Restricted cubic spline (RCS) models with five knots placed at the default percentile locations of the RCS implementation (approximately the 5th, 27.5th, 50th, 72.5th, and 95th percentiles of the ALBI distribution) were fitted to explore potential nonlinear associations. The spline analysis was stratified by sex *a priori* to examine potential sex-specific nonlinear patterns. Subgroup analyses were conducted to explore potential effect modification. Interaction terms were used to evaluate heterogeneity in the association between ALBI score and gallstone prevalence.

To further identify key associated factors and enhance predictive performance, machine-learning models were developed using the NHANES analytic dataset. The dataset was randomly divided into a training set and a held-out internal test set at a ratio of 7:3 using a fixed random seed of 2025. With ten candidate predictors and approximately 343 events in the training split (70% of 490 events), the events-per-predictor ratio is approximately 34, exceeding the commonly recommended minimum of 10–20 events per predictor for binary outcomes. The split was performed before model training, and the internal test set was used only for final model evaluation.

Candidate predictors for the machine-learning models included age, sex, race/ethnicity, poverty income ratio, education level, smoking status, alcohol consumption, diabetes, hypertension, and ALBI score. Cardiovascular disease was not included in the candidate predictor set, reflecting the different purposes of the association and prediction analyses. Fifteen predefined model combinations were evaluated. The fifteen combinations were defined *a priori* to span the main methodological families: LASSO with logistic regression, XGBoost, and SVM; Boruta with random forest and XGBoost; univariate AUC ranking with logistic regression, XGBoost, and SVM; PCA (top five components) with logistic regression, XGBoost, and SVM; random forest importance ranking with logistic regression and SVM; and gradient boosting and k-nearest neighbors each paired with logistic regression. The remaining possible pairings were deliberately not evaluated. The label convention used throughout is “feature-selection method + classifier”; for the GBM + Logistic and KNN + Logistic entries, the feature-ranking step was not applied to select a subset, so they behave as full-feature logistic regression models.

Model selection was performed within the training set using 5-fold cross-validation. Preprocessing and feature-selection steps were conducted within the resampling procedure to reduce information leakage. In preprocessing, continuous variables were standardized, categorical variables were one-hot encoded, and missing data were handled by complete-case exclusion before modeling. Hyperparameters were prespecified before training rather than optimized through an extensive grid-search procedure. The leading configurations based on cross-validated AUC were subsequently evaluated in the held-out internal test set.

Model discrimination was assessed using receiver operating characteristic curves and the area under the curve. Calibration was assessed visually using calibration plots, and decision curve analysis was used to examine model net benefit across threshold probabilities. SHAP analysis was applied to quantify the relative importance and directionality of each feature's contribution to model predictions. The TRIPOD + AI statement (20) and the STROBE statement (21) were used to guide the reporting of the prediction-model and observational components, respectively. External validation was performed using an independent hospital-based retrospective cohort from Zaozhuang Municipal Hospital.

NHANES survey weights were used for population-based association analyses, including descriptive statistics, regression models, restricted cubic spline analyses, and subgroup analyses. Machine-learning models were developed primarily for classification in the analyzed sample; therefore, their AUC, calibration, and decision-curve results should not be interpreted as design-based nationally representative estimates.

## Results

### Baseline characteristics

A total of 5,090 participants were included in this study, with a survey-weighted mean age of 46.81 years; 2,599 (51.19%, survey-weighted) were women and 2,491 (48.81%, survey-weighted) were men. Among them, 490 participants (9.63%, crude proportion) were identified as having gallstones. Participants with and without gallstones differed in age, sex, diabetes, hypertension, cardiovascular disease, and ALBI score (all *P* < 0.05); differences in ethnicity, poverty income ratio, education, smoking, and alcohol consumption did not reach significance. The baseline characteristics of all participants are presented in Supplementary Table S1.

### Association between the ALBI score and gallstones

The association between ALBI score and gallstone prevalence was evaluated by modeling ALBI both as a continuous variable and as a categorical variable. When ALBI was modeled as a continuous variable, higher ALBI score was significantly associated with increased gallstone prevalence across all models. In the fully adjusted model, each 1-unit increase in ALBI score was associated with higher odds of gallstones (Model III: OR = 2.89, 95% CI: 1.68–4.99, *P* = 0.002; Table 1).

**Table 1:** Association between ALBI score and gallstones in different models.

Exposure	Model I		Model II		Model III	
	OR (95% CI)	*P* value	OR (95% CI)	*P* value	OR (95% CI)	*P* value
ALBI score	4.25 (2.78, 6.49)	<0.001	3.08 (1.95, 4.85)	<0.001	2.89 (1.68, 4.99)	0.002
ALBI score, (groups)						
Low-range	ref	—	ref	—	ref	—
Upper-range	2.47 (1.71, 3.57)	<0.001	2.08 (1.40, 3.08)	0.001	2.06 (1.35, 3.15)	0.010

ALBI, albumin-bilirubin; OR, odds ratio; CI, confidence interval; PIR, poverty income ratio; CVD, cardiovascular disease. For the continuous analysis, ORs represent the association between each 1-unit increase in ALBI score and gallstone prevalence. For the categorical analysis, a low-range ALBI was defined as ALBI ≤ −2.60, and an upper-range ALBI was defined as ALBI > −2.60. Model I was unadjusted. Model II was adjusted for age, sex, race/ethnicity, and education level. Model III was further adjusted for poverty income ratio, smoking status, alcohol consumption, diabetes, hypertension, and cardiovascular disease. All models accounted for the NHANES complex survey design using sampling weights, masked variance pseudo-strata, and pseudo-primary sampling units. The design degrees of freedom for the survey-weighted analyses are based on the number of pseudo-strata and pseudo-PSUs in the NHANES 2017–March 2020 cycle.

When ALBI was modeled as a categorical variable, participants with an ALBI score in the upper range (above −2.60) had significantly higher odds of gallstones than those with an ALBI score in the lower range (≤ −2.60). This association remained significant after full adjustment for age, sex, race/ethnicity, education level, poverty income ratio, smoking status, alcohol consumption, diabetes, hypertension, and cardiovascular disease (Model III: OR = 2.06, 95% CI: 1.35–3.15, *P* = 0.010; Table 1).

### Nonlinear association between ALBI and gallstones

Restricted cubic spline (RCS) analyses were performed to evaluate potential nonlinear associations between ALBI score and gallstone prevalence after adjusting for age, ethnicity, PIR, education, smoking, alcohol consumption, diabetes, hypertension, and CVD.

**Figure 1:**
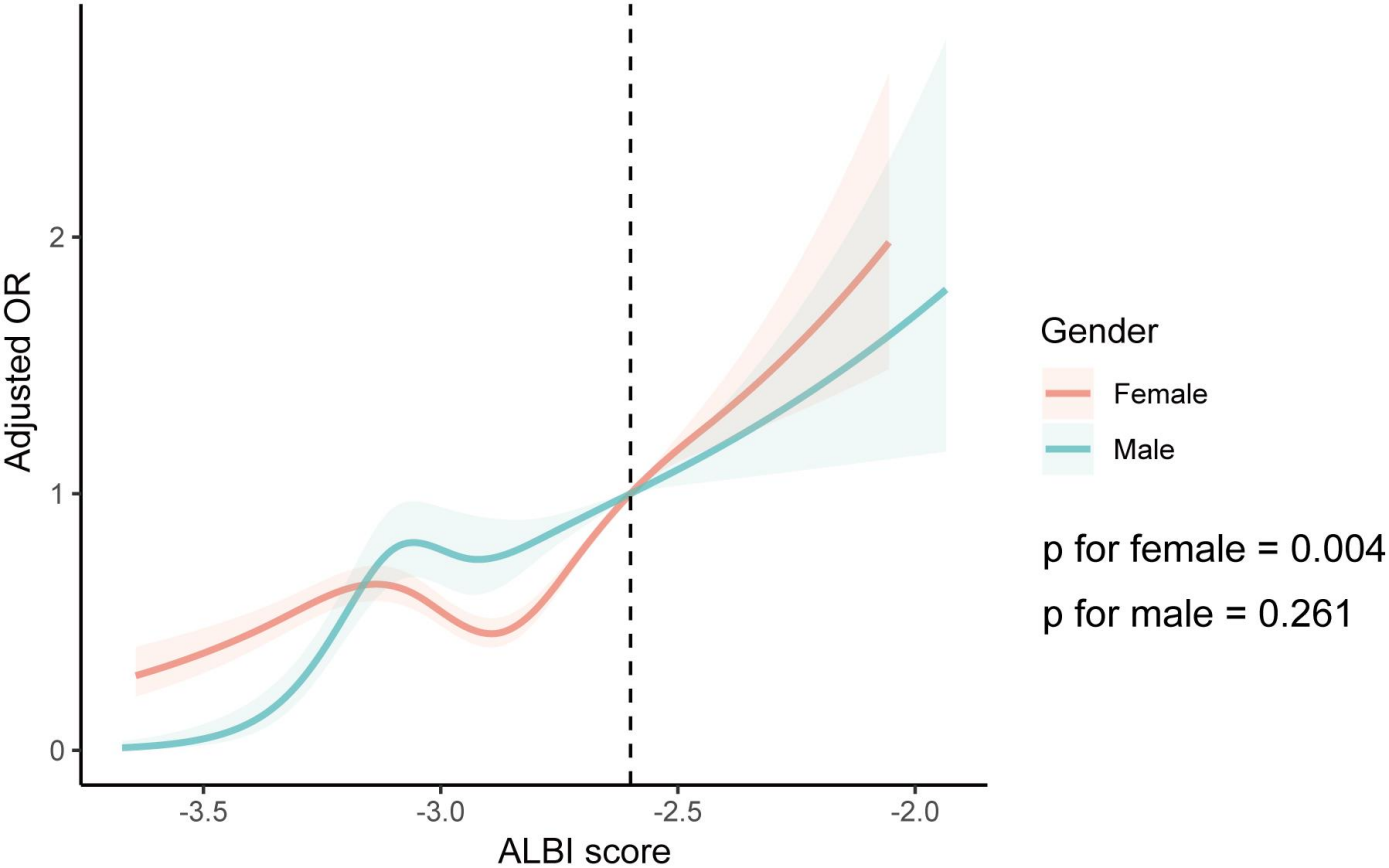
Dose–response relationship between ALBI score and gallstone prevalence. Restricted cubic spline analysis showing the association between ALBI score and gallstone prevalence by sex. The solid line represents the adjusted OR, and the shaded area represents the 95% confidence interval. The reference ALBI value at which the adjusted odds ratio equals one is indicated (the plotted curves cross unity at the −2.60 cut-point). The splines were fitted with five knots placed at approximately the 5th, 27.5th, 50th, 72.5th, and 95th percentiles of the ALBI distribution. The model was adjusted for age, race/ethnicity, PIR, education level, smoking status, alcohol consumption, diabetes, hypertension, and CVD. ALBI, albumin-bilirubin; OR, odds ratio; CI, confidence interval; PIR, poverty income ratio; CVD, cardiovascular disease.

Among men, no significant nonlinear association was observed (*P* for nonlinearity = 0.261), while a significant nonlinear trend was identified in women (*P* for nonlinearity = 0.004). When the ALBI score exceeded the pre-specified cut-point of −2.60, the likelihood of gallstone presence increased approximately linearly in both sexes. The sex-specific nonlinearity tests are displayed in Figure 1 (*P* = 0.004 in women, *P* = 0.261 in men); no global test across both sexes was computed.

### Subgroup analysis

To explore potential effect modification, subgroup analyses were conducted by sex, race/ethnicity, PIR, education level, smoking status, alcohol consumption, diabetes, hypertension, and CVD. In these subgroup analyses, ALBI was modeled as a continuous variable. The positive association between higher ALBI score and gallstone prevalence remained generally consistent across most subgroups.

Notably, a significant interaction was observed for CVD status (*P* for interaction = 0.022). Among participants without CVD, each 1-unit increase in ALBI score was associated with higher odds of gallstones (OR = 4.15, 95% CI: 2.89–5.97, *P* < 0.001), whereas the association was weaker and not statistically significant among participants with CVD (OR = 1.59, 95% CI: 0.76–3.35, *P* = 0.220). Although Non-Hispanic White participants showed the largest effect estimate (OR = 5.39, 95% CI: 3.17–9.18, *P* < 0.001), the interaction across race/ethnicity groups was not statistically significant (*P* for interaction = 0.117). Detailed results are presented in Table 2.

**Table 2:** Subgroup analysis for the association between ALBI score and gallstone prevalence.

Subgroup	OR (95% CI)	*P* value	*P* for interaction
Sex			0.640
Female	3.37 (2.25, 5.06)	<0.001	
Male	3.98 (2.28, 6.95)	<0.001	
Race			0.117
Non-Hispanic White	5.39 (3.17, 9.18)	<0.001	
Mexican American	3.39 (1.48, 7.77)	0.004	
Non-Hispanic Black	3.98 (1.95, 8.15)	<0.001	
Other Hispanic	2.47 (0.91, 6.71)	0.076	
Other Race	5.24 (2.26, 12.18)	<0.001	
PIR			0.231
Low	4.54 (2.55, 8.07)	<0.001	
Medium	2.95 (1.70, 5.12)	<0.001	
High	4.40 (2.55, 7.58)	<0.001	
Education level			0.228
High school and below	4.96 (2.97, 8.27)	<0.001	
Above high school	3.31 (2.19, 5.00)	<0.001	
Smoking status			0.784
Never	3.73 (2.38, 5.84)	<0.001	
Former	3.85 (2.06, 7.22)	<0.001	
Current	4.17 (2.13, 8.16)	<0.001	
Alcohol consumption			0.208
Never	3.22 (1.25, 8.32)	0.016	
Mild	3.27 (2.01, 5.31)	<0.001	
Moderate	5.22 (2.65,10.30)	<0.001	
Heavy	4.58 (2.31, 9.05)	<0.001	
Diabetes			0.545
No	3.69 (2.50, 5.44)	<0.001	
Yes	2.97 (1.65, 5.33)	<0.001	
Hypertension			0.523
No	3.83 (2.39, 6.12)	<0.001	
Yes	3.10 (1.98, 4.84)	<0.001	
CVD			0.022
No	4.15 (2.89, 5.97)	<0.001	
Yes	1.59 (0.76, 3.35)	0.220	

ALBI, albumin-bilirubin; OR, odds ratio; CI, confidence interval; PIR, poverty income ratio; CVD, cardiovascular disease. ORs in subgroup analyses represent the association between each 1-unit increase in continuous ALBI score and gallstone prevalence within each subgroup. Models were adjusted for age, sex, race/ethnicity, PIR, education level, smoking status, alcohol consumption, diabetes, hypertension, and CVD, except for the corresponding stratification variable. Interaction effects were tested by adding cross-product terms between ALBI score and subgroup variables. Survey weights, strata, and clusters were incorporated in all subgroup analyses. The stratum-specific estimates are presented for exploratory purposes; their confidence intervals are wide and should not be over-interpreted as indicating stronger effects than the overall estimate, owing to smaller stratum-specific sample sizes, reduced numbers of events within strata, and the non-collapsibility of the odds ratio.

### Machine learning–based classification modeling

To evaluate the classification utility of the albumin–bilirubin (ALBI) score for identifying participants with gallstones, a machine-learning framework was established using NHANES data. As described in the Methods, the dataset was randomly divided into a training set and a held-out internal test set. Fifteen combinations of classical feature selection and classification algorithms were developed and assessed through cross-validation, with the mean AUCs summarized in Supplementary Figure S2.

Among all model configurations, the top-performing models showed essentially equivalent discriminative performance, with differences well within the expected variability of the cross-validation procedure. The Random Forest (RF) + Logistic model achieved the highest cross-validated AUC (0.720), with the other leading configurations reaching 0.718–0.719; these differences should be regarded as indistinguishable.

### Internal validation and model interpretability

**Figure 2:**
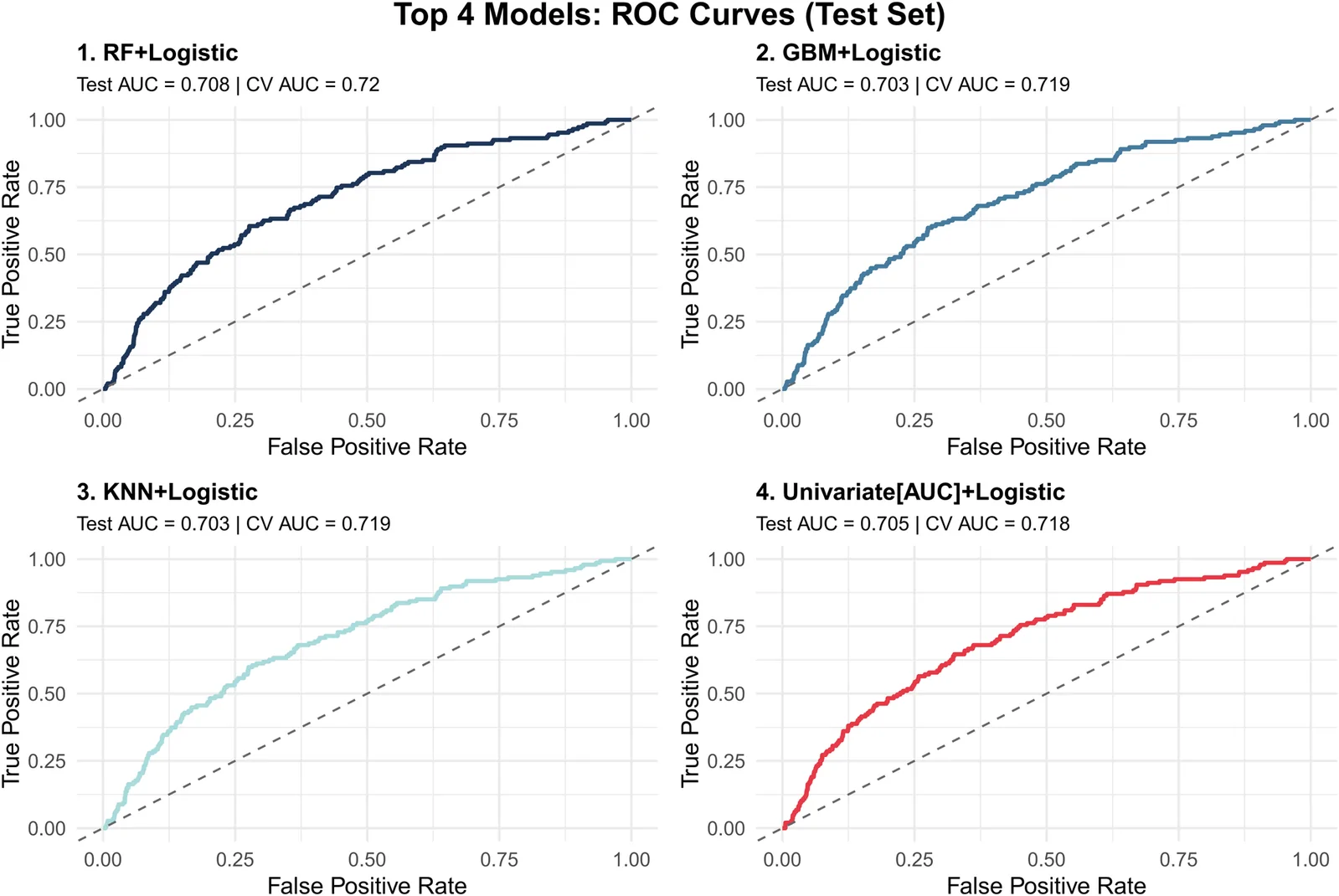
ROC curves of the top four machine-learning models in the internal test set. Receiver operating characteristic curves of the four best-performing model combinations in the held-out internal test set. The RF + Logistic model showed the highest AUC among the evaluated models, indicating moderate discrimination, followed by Univariate AUC + Logistic, with GBM + Logistic and KNN + Logistic showing slightly lower and similar AUCs. RF, random forest; GBM, gradient boosting machine; KNN, k-nearest neighbors; AUC, area under the receiver operating characteristic curve; ROC, receiver operating characteristic.

Model performance was further evaluated using the held-out internal test set. The RF + Logistic model again achieved the highest test AUC (0.708), with cross-validated AUC of 0.720 in the training phase. Comparable test AUCs were obtained for the GBM + Logistic (0.703), KNN + Logistic (0.703), and Univariate AUC + sLogistic (0.705) models (Figure 2), suggesting moderate discriminative performance across the top configurations.

**Figure 3:**
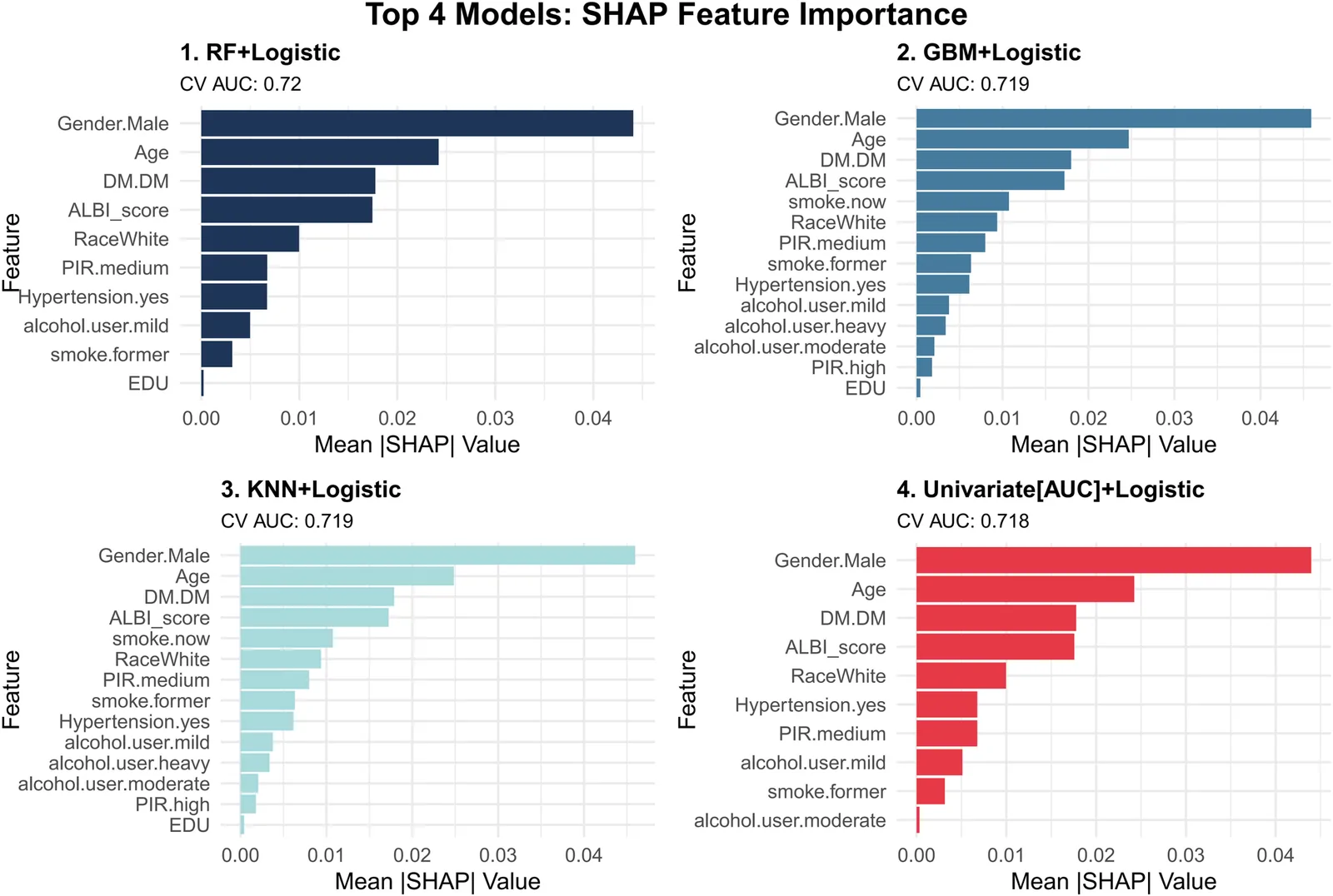
SHAP feature-importance plots of the top four machine-learning models. SHAP feature-importance plots of the top four model combinations, including RF + Logistic, GBM + Logistic, KNN + Logistic, and Univariate AUC + Logistic. SHAP analysis was used to evaluate the relative contribution and directionality of each predictor to model predictions. RF, random forest; GBM, gradient boosting machine; KNN, k-nearest neighbors; AUC, area under the receiver operating characteristic curve; SHAP, SHapley Additive exPlanations; ALBI, albumin-bilirubin; DM, diabetes mellitus; PIR, poverty income ratio.

To enhance interpretability, SHAP (SHapley Additive exPlanations) analysis was performed to quantify the relative contribution of each variable in the internal validation analysis (Figure 3). ALBI score was the fourth-ranked predictor behind sex, age, and diabetes, supporting its contribution to model-based classification of gallstone status.

Calibration was assessed visually using calibration plots (Supplementary Figure S3). Decision curve analysis (DCA) was performed by comparing the model curves with the “treat-all” and “treat-none” reference strategies across the evaluated threshold probabilities (Supplementary Figure S4).

### External validation

In the hospital-based external validation cohort, 500 participants were included, including 42 participants with gallstones and 458 participants without gallstones. The gallstone prevalence was 8.4%. Baseline characteristics of the external validation cohort are shown in Supplementary Table S2. Similar to the NHANES cohort, participants with gallstones in the hospital cohort were older, more likely to be female, and had higher ALBI scores than those without gallstones. However, diabetes, hypertension, and CVD did not differ significantly between gallstone and non-gallstone groups in the hospital cohort, possibly because of the smaller sample size and fewer gallstone cases. The small number of events (42) in the external cohort means that these results provide only preliminary evidence of generalizability and cannot be interpreted as demonstrating stable or definitive model performance.

**Figure 4:**
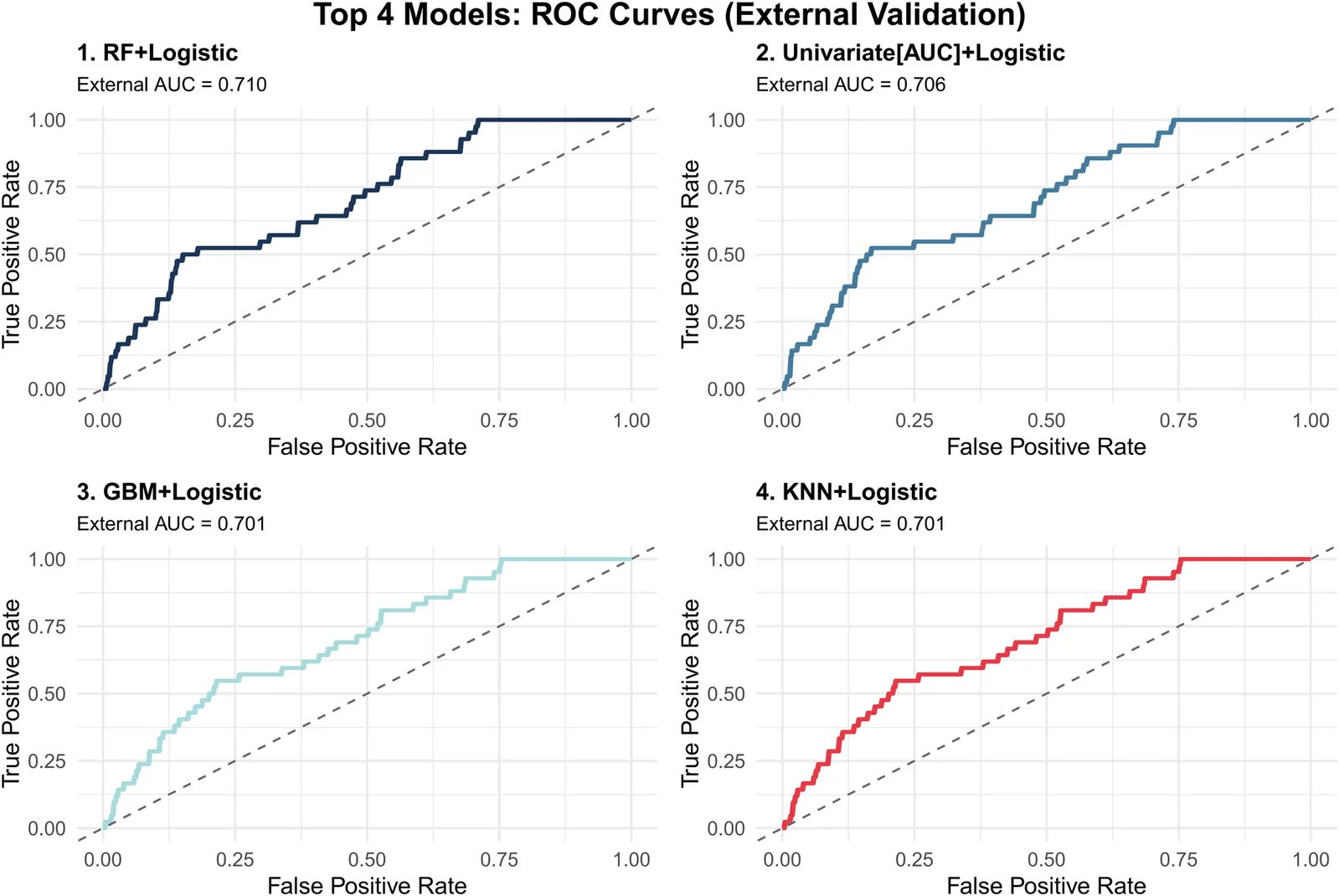
ROC curves of the top four machine-learning models in the external validation cohort. Receiver operating characteristic curves of the four best-performing machine-learning model combinations in the independent hospital-based external validation cohort. The RF + Logistic model achieved the highest AUC of 0.710, followed by Univariate AUC + Logistic with an AUC of 0.706, GBM + Logistic with an AUC of 0.701, and KNN + Logistic with an AUC of 0.701. RF, random forest; GBM, gradient boosting machine; KNN, k-nearest neighbors; AUC, area under the receiver operating characteristic curve; ROC, receiver operating characteristic.

The hospital-based external validation cohort consisted entirely of Chinese participants (single ethnicity), so that the race/ethnicity term takes a single constant value. External validation using this cohort yielded AUC values ranging from 0.701 to 0.710 across the top-performing models. The RF + Logistic model achieved the highest AUC (0.710), followed by Univariate AUC + Logistic (0.706), GBM + Logistic (0.701), and KNN + Logistic (0.701) (Figure 4). However, the 95% confidence intervals around these estimates are wide (approximately 0.62–0.80) owing to the small number of events (*n* = 42), and these external results provide only preliminary evidence of generalizability.

In the external validation cohort, SHAP analysis further identified ALBI score as an important predictor, ranking second among the displayed predictors across all top-performing models. The internal and external rankings are reported separately to allow the reader to assess the difference across cohorts (Supplementary Figure S5).

## Discussion

In this analysis of nationally representative NHANES data, further evaluated using an independent Chinese hospital-based validation cohort, we identified a significant positive association between higher ALBI scores and the presence of gallstones. In the fully adjusted model, continuous ALBI score was positively associated with gallstone prevalence (OR = 2.89, 95% CI: 1.68–4.99, *P* = 0.002). In the categorical analysis using the pre-specified −2.60 cut-point, participants with an ALBI score in the upper range (above −2.60) showed higher odds of gallstones compared with those with an ALBI score in the lower range (≤ −2.60) (OR = 2.06, 95% CI: 1.35–3.15, *P* = 0.010). These findings suggest that elevated ALBI scores are associated with gallstone status. The ALBI score may reflect hepatic synthetic/excretory function, systemic inflammation, or frailty.

The ALBI composite was selected as the primary exposure because it is a validated, widely used summary of hepatic synthetic and excretory function that captures the joint state of its two components, and composite scores of this type are routinely used in hepatology. However, albumin and total bilirubin may have distinct biological relationships to lithogenesis, so component-specific associations cannot be excluded. Separate analyses of albumin and total bilirubin with mutual adjustment would therefore be informative in future work. The NHANES 2017–March 2020 gallstone item has been used in several recent analyses examining anthropometric and metabolic exposures and prediction approaches (22–26). Separately, ALBI has also been evaluated in NHANES in relation to sarcopenia (27). The genuinely novel element of the present manuscript is the specific pairing of the ALBI score with gallstone disease, which, to the best of our knowledge, has not previously been reported.

The ALBI score is a well-established measure for assessing hepatic functional reserve. Compared with conventional scoring systems such as the Child–Pugh and MELD scores, the ALBI score is simpler and more objective, as it relies solely on two routine biochemical parameters—serum albumin and total bilirubin—without including subjective components such as ascites or encephalopathy (28, 29). Albumin, the most abundant plasma protein synthesized by the liver, reflects both hepatic synthetic function and nutritional status; low levels are often indicative of hepatic dysfunction or systemic inflammation (30). Bilirubin, a product of heme degradation, reflects hepatic metabolic and excretory capacity; elevated levels are associated with hepatocellular injury and cholestasis (31).

In a community-based sample with little overt liver disease, the ALBI score should not be interpreted exclusively as a measure of hepatic reserve. Albumin is a negative acute-phase protein whose circulating concentration may decrease with inflammation and is also influenced by nutritional status (32). The observed association is therefore compatible with two non-exclusive interpretations: (i) reduced hepatic synthetic/excretory function, and (ii) systemic inflammation, malnutrition, or frailty, which lower albumin independently of the liver. The association between ALBI and gallstone prevalence may reflect, at least in part, the latter pathway rather than hepatic functional reserve alone.

Previous studies have clarified the biological importance of these two parameters in gallstone formation. Li et al. (33) demonstrated that monoconjugated bilirubin may act as a coprecipitant with unconjugated bilirubin during gallstone nucleation (33), while bilirubin has also been reported to contribute to both pigment and cholesterol stone formation by influencing bile lithogenicity (34, 35). Although albumin is not directly involved in stone nucleation, it participates in lipid transport and influences bile cholesterol saturation—key processes in cholesterol gallstone formation (32). Moreover, albumin is involved in calcium homeostasis, and calcium–bilirubinate complexes can promote gallstone crystallization (36). Collectively, these mechanisms provide biological plausibility for an association between the ALBI score and gallstone presence, while not establishing a causal pathway.

The positive association between higher ALBI score and gallstone prevalence was generally observed across the reported sex, ethnicity, socioeconomic, lifestyle, and metabolic subgroups. Notably, CVD showed a significant interaction effect (*P* for interaction = 0.022), with a stronger association observed in individuals without CVD (OR = 4.15) than in those with CVD (OR = 1.59). This subgroup finding is presented as a hypothesis-generating observation and was one of multiple interactions tested; it would not be considered significant after correction for multiple testing. No mechanistic interpretation is proposed for this interaction. Residual confounding and limited statistical power within subgroups cannot be fully excluded. The restricted cubic spline analyses further indicated a nearly linear increase in gallstone prevalence when the ALBI score exceeded −2.60, consistent with the pre-specified analytic cut-point.

In addition to conventional regression analyses, machine-learning techniques were applied to explore whether the ALBI score and related clinical variables could help identify individuals with gallstones. By integrating multiple feature-selection and classification algorithms, the Random Forest-based feature-selection method combined with logistic regression achieved the highest cross-validated AUC (0.720), although the differences between configurations were small and within the expected variability of the cross-validation procedure. Overall, the leading configurations yielded similar moderate discrimination, and the small differences between them should not be interpreted as evidence of a clinically meaningful advantage of any one modeling strategy. External validation in an independent hospital-based retrospective cohort showed moderate discriminatory performance, with AUC values ranging from 0.701 to 0.710 across the top-performing configurations. SHAP analysis identified the ALBI score as an important predictor in both internal and external analyses, supporting its contribution to gallstone classification. The transportability of the findings should be interpreted cautiously, particularly given the asymmetric exclusion criteria between the two cohorts. The hospital cohort excluded viral hepatitis, cirrhosis, hepatocellular carcinoma, and malignancy, whereas the NHANES sample did not. This asymmetry is explained by the different study purposes: the hospital cohort was restricted to the target population of community-dwelling adults without known advanced liver disease, whereas in NHANES such conditions are rare and the corresponding exclusion could not be applied. Differences in ethnicity, healthcare setting, referral patterns, disease spectrum, predictor distributions, and gallstone ascertainment between the two cohorts should be considered when interpreting the results.

Several limitations should be considered. First, because the NHANES analysis was cross-sectional and the hospital-based validation cohort was retrospective, causality cannot be inferred. Reverse causation is a particular concern: choledocholithiasis, biliary sludge, prior cholecystitis, and subclinical cholestasis can all raise total bilirubin, while symptomatic biliary disease can lower albumin through systemic inflammation. The observed association could therefore reflect, at least in part, the effect of gallstone-related biliary pathology on the ALBI components. Second, gallstone status in NHANES was based on self- or proxy-reported physician diagnosis rather than imaging confirmation, which may have introduced recall bias, under-ascertainment of asymptomatic gallstones, and outcome misclassification. In contrast, gallstone status in the hospital-based validation cohort was confirmed by abdominal ultrasonography or surgical findings; differences in outcome ascertainment between the two cohorts should therefore be considered when interpreting the results. Third, complete-case analysis may have introduced selection bias, and residual confounding from unmeasured factors such as diet, genetic predisposition, medication use, and metabolic profiles cannot be excluded. Residual confounding by body size (BMI, waist circumference) and lipid profile also cannot be fully excluded. Future prospective studies with imaging-confirmed gallstone outcomes, including age-stratified analyses, are warranted.

## Conclusion

In a national sample of US adults, a higher ALBI score remained associated with higher odds of gallstone disease after multivariable adjustment. Machine-learning models incorporating ALBI achieved moderate discrimination, with preliminary external-validation support that requires confirmation in larger cohorts. Future prospective studies with imaging-confirmed gallstone outcomes, adequate sample sizes for external validation, and adjustment for body-size measures are needed to clarify the temporal and causal relationships and to determine whether ALBI provides information beyond established risk factors.

## Data Availability

The original contributions presented in the study are included in the article/Supplementary Material, further inquiries can be directed to the corresponding author/s.
