# Supplementary material for: Association between the albumin–bilirubin score and gallstone prevalence: evidence from NHANES and an independent hospital-based cohort
Source: Front Med (Lausanne). 2026 Sep 10;13:1872983. doi: 10.3389/fmed.2026.1872983 (PMC13600880; doi:10.3389/fmed.2026.1872983)
Supplement: Supplementary file 1 [file Datasheet1.docx]

**Supplementary Table S1. Baseline characteristics of participants**

| Variable | **Total** | No gallstones | **Gallstones** | **P value** |
| --- | --- | --- | --- | --- |
| Age | 46.81(0.69) | 45.98(0.68) | 54.67(1.05) | <0.001 |
| Sex, n (%) |  |  |  | <0.001 |
| Female | 2599(51.19) | 2243(48.48) | 356(77.02) |  |
| Male | 2491(48.81) | 2357(51.52) | 134(22.98) |  |
| Race, n (%) |  |  |  | 0.149 |
| Non-Hispanic White | 1889(65.91) | 1673(65.45) | 216(70.30) |  |
| Mexican American | 601(8.12) | 533(8.08) | 68(8.46) |  |
| Non-Hispanic Black | 1252(10.07) | 1168(10.48) | 84(6.20) |  |
| Other Hispanic | 493(6.74) | 440(6.74) | 53(6.73) |  |
| Other Race | 855(9.16) | 786(9.25) | 69(8.30) |  |
| PIR, n (%) |  |  |  | 0.090 |
| Low | 1327(16.62) | 1200(16.70) | 127(15.83) |  |
| Medium | 1633(26.40) | 1464(25.88) | 169(31.33) |  |
| High | 2130(56.99) | 1936(57.42) | 194(52.84) |  |
| Education level, n (%) |  |  |  | 0.236 |
| High school and below | 1917(33.84) | 1724(33.56) | 193(36.51) |  |
| Above high school | 3173(66.16) | 2876(66.44) | 297(63.49) |  |
| Smoking status, n (%) |  |  |  | 0.055 |
| Never | 2997(58.95) | 2735(59.52) | 262(53.48) |  |
| Former | 1160(25.10) | 1021(24.48) | 139(31.04) |  |
| Current | 933(15.95) | 844(16.00) | 89(15.48) |  |
| Alcohol consumption, n (%) |  |  |  | 0.053 |
| Never | 532(7.46) | 471(7.11) | 61(10.87) |  |
| Mild | 2303(45.46) | 2072(45.36) | 231(46.45) |  |
| Moderate | 1081(22.61) | 976(22.50) | 105(23.62) |  |
| Heavy | 1174(24.47) | 1081(25.04) | 93(19.06) |  |
| Diabetes, n (%) |  |  |  | <0.001 |
| No | 4141(85.99) | 3803(87.08) | 338(75.58) |  |
| Yes | 949(14.01) | 797(12.92) | 152(24.42) |  |
| Hypertension, n (%) |  |  |  | <0.001 |
| No | 3269(69.51) | 3026(71.02) | 243(55.09) |  |
| Yes | 1821(30.49) | 1574(28.98) | 247(44.91) |  |
| CVD, n (%) |  |  |  | <0.001 |
| No | 4562(91.59) | 4166(92.14) | 396(86.31) |  |
| Yes | 528(8.41) | 434(7.86) | 94(13.69) |  |
| ALBI score | −2.94(0.01) | −2.95(0.01) | −2.85(0.02) | <0.001 |

Continuous variables are presented as survey-weighted means with standard errors, and categorical variables as unweighted counts with survey-weighted percentages. PIR: Poverty income ratio; CVD: Cardiovascular disease; ALBI score: Albumin-Bilirubin score.

**Supplementary Table S2. Baseline characteristics of the hospital-based external validation cohort**

| **Characteristic** | **Overall (N = 500)** | **No gallstones (n = 458)** | **Gallstones (n = 42)** | **P value** |
| --- | --- | --- | --- | --- |
| Age, years | 46.38 ± 9.81 | 45.79 ± 9.64 | 52.79 ± 9.44 | <0.001 |
| ALBI score | −2.93 ± 0.39 | −2.95 ± 0.38 | −2.75 ± 0.43 | 0.006 |
| Sex |  |  |  | 0.018 |
| Female | 252 (50.4) | 223 (48.7) | 29 (69.0) |  |
| Male | 248 (49.6) | 235 (51.3) | 13 (31.0) |  |
| PIR / income level |  |  |  | 0.898 |
| Low | 104 (20.8) | 95 (20.7) | 9 (21.4) |  |
| Medium | 195 (39.0) | 180 (39.3) | 15 (35.7) |  |
| High | 201 (40.2) | 183 (40.0) | 18 (42.9) |  |
| Education level |  |  |  | 1.000 |
| High school and below | 188 (37.6) | 172 (37.6) | 16 (38.1) |  |
| Above high school | 312 (62.4) | 286 (62.4) | 26 (61.9) |  |
| Smoking status |  |  |  | 0.298 |
| Never | 356 (71.2) | 328 (71.6) | 28 (66.7) |  |
| Former | 79 (15.8) | 69 (15.1) | 10 (23.8) |  |
| Current | 65 (13.0) | 61 (13.3) | 4 (9.5) |  |
| Alcohol consumption |  |  |  | 0.103 |
| Never | 297 (59.4) | 276 (60.3) | 21 (50.0) |  |
| Mild | 148 (29.6) | 134 (29.3) | 14 (33.3) |  |
| Moderate | 41 (8.2) | 34 (7.4) | 7 (16.7) |  |
| Heavy | 14 (2.8) | 14 (3.1) | 0 (0.0) |  |
| Diabetes |  |  |  | 1.000 |
| No | 446 (89.2) | 408 (89.1) | 38 (90.5) |  |
| Yes | 54 (10.8) | 50 (10.9) | 4 (9.5) |  |
| Hypertension |  |  |  | 0.109 |
| No | 357 (71.4) | 332 (72.5) | 25 (59.5) |  |
| Yes | 143 (28.6) | 126 (27.5) | 17 (40.5) |  |
| CVD |  |  |  | 0.069 |
| No | 459 (91.8) | 424 (92.6) | 35 (83.3) |  |
| Yes | 41 (8.2) | 34 (7.4) | 7 (16.7) |  |
| ALBI category |  |  |  | 0.016 |
| Low-range (≤ −2.60) | 399 (79.8) | 372 (81.2) | 27 (64.3) |  |
| Upper-range (> −2.60) | 101 (20.2) | 86 (18.8) | 15 (35.7) |  |

Continuous variables are presented as mean ± SD, and categorical variables are presented as n (%). P values were calculated using Welch’s t-test for continuous variables and chi-square or Fisher’s exact tests for categorical variables, as appropriate. ALBI, albumin-bilirubin; PIR, poverty income ratio; CVD, cardiovascular disease.

**Figure S1. Flow chart of participant selection. NHANES, National Health and Nutrition Examination Survey**

Figure S2. Comparison of cross-validated AUC across feature-selection and model combinations. Comparison of cross-validated area under the ROC curve (AUC) values obtained from different feature-selection and classification model combinations. Each bar represents the mean AUC from 5-fold cross-validation.

Figure S3. Calibration curves of the top four models in the held-out internal test set. The dashed diagonal line represents perfect calibration. The plots show the degree of agreement between predicted and observed risk across the probability range.

Figure S4. Decision curve analysis (DCA) of the top four models in the held-out internal test set. The model curves are shown relative to the “treat-all” and “treat-none” reference lines across the evaluated threshold probabilities.

Figure S5. SHAP feature-importance plots of the top four machine-learning models in the external validation cohort. In the external cohort, ALBI ranked second among the displayed predictors across all top-performing models. SHAP, SHapley additive exPlanations; RF, random forest; GBM, gradient boosting machine; KNN, k-nearest neighbors; ALBI, albumin-bilirubin.

**Supplementary Table S3. Mapping of alcohol consumption and socioeconomic categories across cohorts**

A. Alcohol consumption categories

| Category | NHANES | Hospital cohort |
| --- | --- | --- |
| Never | No alcohol consumption (0 drinks/day) | 0 g/day |
| Mild | ≤1 (women) / ≤2 (men) drinks/day | ≤12 g/day |
| Moderate | 2–3 (women) / 3–4 (men) drinks/day | >12–36 g/day |
| Heavy | ≥4 (women) / ≥5 (men) drinks/day | >36 g/day |

Note: The cut-points are not fully equivalent across cohorts. In NHANES, alcohol consumption is based on self-reported drinks per day; in the hospital cohort, intake is based on grams of pure alcohol per day. The mild category corresponds to up to ~14 g/day for women and ~28 g/day for men in NHANES (assuming 14 g/drink), compared with 12 g/day in the hospital cohort.

B. Socioeconomic status categories

| Category | NHANES (PIR) | Hospital cohort (income) |
| --- | --- | --- |
| Low | <1.3 | Low monthly household income band |
| Medium | ≥1.3 to <3.0 | Medium monthly household income band |
| High | ≥3.0 | High monthly household income band |

Note: Socioeconomic status is represented by the poverty income ratio (PIR) in NHANES and by predefined income bands in the hospital cohort. The categories are not directly equivalent; PIR is a ratio of family income to the federal poverty threshold, whereas the hospital cohort used predefined monthly household income bands. The exact institutional cut-points were not available in the analytic dataset.
